# Neurasthenia

**Published:** 1910-03

**Authors:** 


**Neurasthenia.** By GILBERT BALLET, M.D. Translated from the third French edition by P. CAMPBELL SMITH, M.D. Pp. xxviii, 408. London : Hy. Kimpton. 1908.

This translation renders accessible to English readers a book which has deservedly attained a high reputation in France. It is the outcome of a wide experience and, what is of equal importance in dealing with this subject, of a sound judgment. The various states grouped under the generic term of neurasthenia are so common in all kinds of practice, that help is frequently required both as to diagnosis and treatment. If the diagnosis presents numerous pitfalls, treatment no less often ends in a confession of failure on the part of the much-tried medical attendant. This volume may be consulted with confidence for a sound and rational exponence of the clinical features of neurasthenia, and the section on treatment, which occupies more than half the book, will be found especially valuable. The translation is well done, and preserves the lucidity of the original work.

